# Acute coronary syndrome secondary to allergic coronary vasospasm (Kounis Syndrome): a case series, follow-up and literature review

**DOI:** 10.1186/s12872-018-0781-9

**Published:** 2018-02-27

**Authors:** Jing Li, Jingang Zheng, Yifeng Zhou, Xiaofei Liu, Wenhua Peng

**Affiliations:** Department of Cardiology, China-Japan Friendship Hospital, No. 2, Yinghua Road, Beijing, 100029 China

**Keywords:** Kounis Syndrome, Anaphylaxis, Coronary vasospasm, Cardiac attack

## Abstract

**Background:**

Kounis syndrome (KS) is the concurrence of acute coronary syndrome associated with mast-cell and platelet activation in the setting of hypersensitivity and allergic or anaphylactic insults. Many drugs and environmental exposures had been reported as inducers, but various inducers and the mechanism of KS remained unknown till now. The widely used traditional Chinese medicine (TCM) as a potential sensitizer were scarcely reported to induce allergic vasospasm due to the ignorance of the linkage between traditional medicine allergy and vasospasm.

**Case presentation:**

We described 5 rare cases of KS including unreported triggers of TCM and abortion, reported the treatment strategy and 1~4 years’ follow-up results, and tried to probe into the etiology of KS. Case 1 and case 2 for the first time reported acute ST-segment elevation myocardial infarction (STEMI) caused by Chinese herbs related allergic coronary vasospasm. Case 3 reported recurrent angina following allergen contact and wheezing, indicating the internal linkage of coronary vasospasm and allergic asthma. Case 4 described a childbearing-age woman suffered refractory ischemic chest pain due to coronary vasospasm in a special period of post-abortion, the attacks suddenly disappeared when her menopause recovered. Case 5 described an isolated episode of allergic coronary vasospasm under exposure of smoking and stress, which was successfully prevented by avoiding the exposures.

**Conclusion:**

Kounis syndrome is not rare but rarely recognized and under-diagnosed. It is necessary to recognize KS and various inducers, especially for the patients suffering refractory vasospastic cardiac attacks concentrating in special periods. Blood test of eosinophil might contribute to diagnose KS and anti-allergic agents might be helpful for controlling KS attacks.

## Background

KS was initially defined as the acute coronary syndrome (angina and myocardial infarction) associated with allergic reaction in 1991 [1]. After that, over 300 cases of KS had been described, researchers emphasizing the existence and association of this syndrome with coronary inflammation and vasospasm. In 2016, Dr. Kounis revised the definition of KS as the concurrence of acute coronary syndrome associated with mast-cell and platelet activation in the setting of hypersensitivity and allergic or anaphylactic insults [2]. Till now, diagnosis of KS is based on clinical manifestations. Many cases may be missed or underdiagnosed due to the unawareness of physicians, determining the prevalence or true incidence of KS is difficult with ore current state of knowledge. Helbling et al. reported an incidence of 7.9-9.6 per 100,000 inhabitants of anaphylaxis with circulatory symptoms per year, and the case-fatality rate was 0.0001% [3], the incidence of KS in the emergency department among all admissions and allergy patients was 19.4 per 100,000 and 3.4%, respectively [1]. Recent reports have shown that KS has been observed in every race, age group (from 9 to 90-year-old) and geographic location, the most common affected age is 40-70 years old (68%). Risk factors of KS include history of previous allergy, hypertension, smoking, diabetes and hyperlipidemia. The number of causes that have been implicated to induce KS is increasing rapidly; of the various identified triggers, the most common triggers were antibiotics (27.4%) and insects’ bites (23.4%) [4], yet more triggers are still beyond detection.

KS is recognized to be “not rare but underdiagnosed”, new triggers are continuously being reported, while unrecognized triggers or circumstances that might induce KS are believed to be identified. We recognized 5 cases of KS induced by different allergens or circumstance, especially TCM. TCM is believed to have few side effects by a large amount of people in China. However, Chinese materia medica (CMM) derived from serious plants, animal components and other sources might contain bioactivators and cause anaphylaxis. Here we report three common CMM: Ma-Huang (plant of ephedra), Di-Long (dried earthworm) and injection cervus and cucumis polypeptide (CCP, the combined extracts from deer horn and sweet melon seeds), which could induce KS.

## Methods

From March, 2011 to January, 2018, we recognized 8 cases of highly suspected KS in the admitted patients of cardiology department of China-Japan Friendship Hospital and collected the data during hospitalization. After discharge, for the first year, the patients were followed up by one cardiologist (the first author of this report) in the cardiology clinic every month, their symptoms, physical examination and ECG were collected. After the first year, the patients were followed every 3 months till January, 2018. Besides these follow-up time, patients could inform the cardiologist of the onset of ischemic chest pain by cell phone. 3 of the 8 patients were lost of follow-up after one year while 5 patients were intact followed up.

## Case presentation

### Case 1

**Fig. 1:**
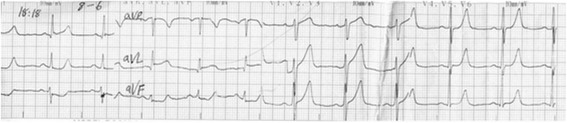
The first ECG demonstrated ST segment elevation of 2 mm in leads I, aVL, and ST depression in leads II, III and aVF

**Fig. 2:**
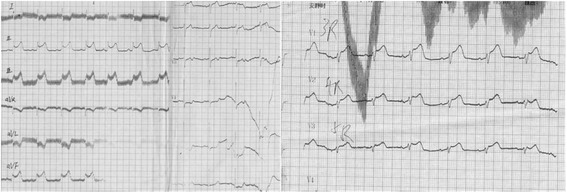
ECG at 20:30 revealed ST elevation of 3-4 mm in leads II, III, aVF, V3R~V5R with a specular changes in leads I, aVL, V4~V6

**Fig. 3:**
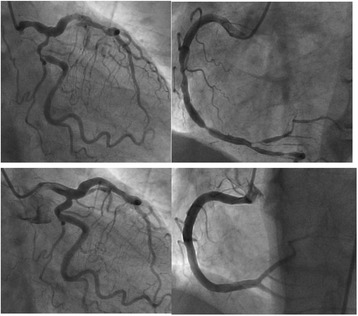
Coronary angiography of: Upper left: left coronary artery spasm; Lower left: left coronary artery relaxed after intracoronary nitroglycerin; Upper right: right coronary artery spasm; Lower right: right coronary artery relaxed after intracoronary nitroglycerin

**Fig. 4:**
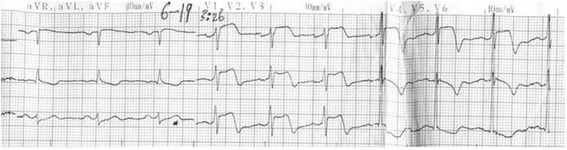
ECG at 3:26on the ambulance revealed ST segment elevation of 4mm in leads V1~V4 and ST depression of 3 mm in leads V5 and V6

A 56 year-old male without known cardiac risk factors presented to the emergency room complaining of retrosternal chest pain radiating to his back with excessive sweating, vertigo and mild dyspnea for about 30 minutes. Physical examination revealed scattered wheezing and a little shortness of breath. The first electrocardiogram (ECG) (18:18) demonstrated ST segment elevation of 2 mm in leads I, aVL, and ST depression in leads II, III and aVF (Fig. 1). The second ECG at 18:32 indicated that all ST segments recovered to baseline. 2 hours later, the chest pain relapsed, ECG showed ST segment elevation of 3-4mm in leads II, III, aVF, V3R~V5R (Fig. 2). Emergency coronary angiography revealed 50% stenosis in the middle segment of left anterior descending(LAD), 50% stenosis in the proximal segment of LCX, 90% stenosis in the middle and subocclusion in the distal segment of right coronary artery (RCA) all the stenosis disappeared after 200μg intracoronary nitroglycerin (Fig. 3). He was diagnosed as coronary vasospasm and given oral isosorbidemononitrate and diltiazem as well as atrovastatin and double anti-platelet therapy (DAPT), chest pain seemed to be controlled. 11 days later, severe substernal chest pain attacked again at 2:40 during sleep, ECG showed ST segment elevation of 4mm in leads V1~V4 and ST depression of 3mm in leads V5 and V6 (Fig. 4). The frequent onset of chest pain was not controlled by doubled anti-vasospasm drugs during hospitalization.

**Fig. 5:**
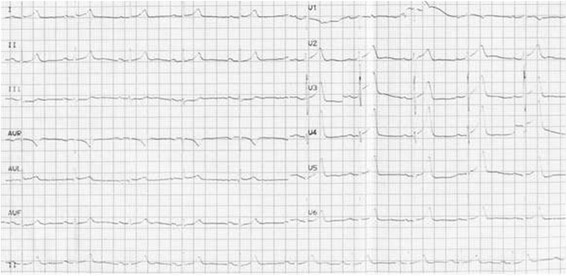
Tall and peaked T waves in leads V3~V6

The frequent onset of ischemic chest pain pushed us to probe into the inducer of coronary vasospasm, and detailed medical history enquiry provided new information pointing to anaphylaxis: the patient had experienced bronchial asthma for 6 months. Seven days before the first attack of cardiac event, he was given Chinese traditional decoction called “Ma-Xing shi gan tang”, which was boiled with Ephedra (Ma-Huang), earthworm (Di-Long) and other herbs. He suffered skin allergy with flushing, itching, erythematous rashes and urticaria on the exact day taking the decoction and experienced several episodes of mild chest pain. Therefore, discontinuation of the traditional herbs therapy (which was the suspicious sensitizer of coronary vasospasm) accompanied by oral loratadine were performed to cure anaphylaxis, this treatment strategy seemed to be effective because the onset of chest pain turned rare and mild in the following 1 month, and the patient kept symptomless for the next 2 months. However, he then attempted to resume the herbs and suffered the 3^rd^ cardiac event, presenting extremely strong chest pain, tall and peaked T waves in leads V3~V6 in ECG (Fig. 5) and obvious elevation of blood eosinophil of 23.4%. This time he completely discarded the herbs and complied to anti-vasospasm therapy for 6 months, he felt no more attacks and discontinued the anti-vasospasm drugs. During the 3 years’ follow-up, he kept free of ischemic chest pain without any anti-vasospasm drugs, although wheezing still relapsed intermittently.

### Case 2

**Fig. 6:**
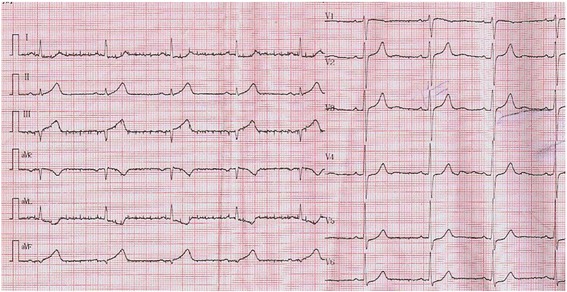
ECG in the local hospital revealed ST segment elevation of 0.5 mm and peaked T wave in leads II, III and avF

**Fig. 7:**
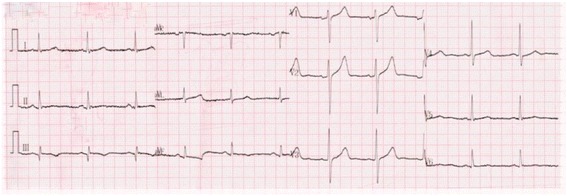
ECG on arrival in our hospital revealed ST segment recovered to baseline in leads II, III and avF and Q wave in lead III

**Fig. 8:**
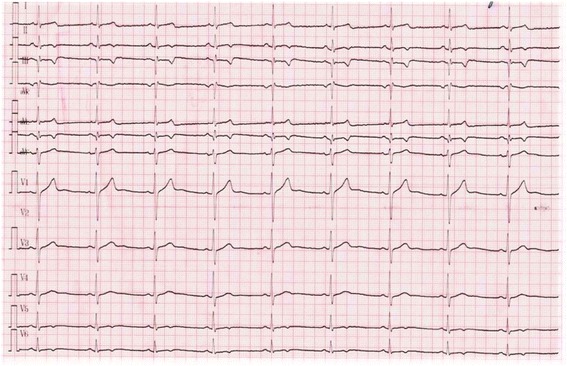
ECG showed ST segment and T wave evolution in leads II, III and avF

A 57 year-old male suffered anaphylactic shock and loss of consciousness when Given Intravenous administration of cervus and cucumis polypeptide for treatment of low back pain in a local hospital. His vital signs recovered in 30 minutes after successful salvage, but then he developed tightening central chest pain and dyspnea, the ECG showed ST segment elevation of 0.5 mm in leads II, III and avF (Fig. 6). He was suspected acute myocardial infarction and sent to our hospital. He had no cardiac risk factors and history of cardiovascular disease (CVD). On admission, his symptom had partly relieved, ECG showed ST segment recovered and Q wave in leads III (Fig. 7). Emergency coronary angiography (5 hours after the event) showed 50% stenosis in the proximal and middle segments of LAD, 25–50% stenosis in the middle segment of LCX and 25–50% stenosis in the middle segment of Without evidence of thrombosis or trace of dissolved thrombosis in the coronary arteries. Chest pain did not relapse during hospitalization, T waves inversion persisted in leads II, III and Q wave in leads III in subsequent ECGs (Fig. 8). His troponin I was 2.27ng/mL on the 5^th^ hour of onset and reached to the peak of 3.0 ng/mL on the 18th hour, and blood eosinophil was 15.5% on arrival. Echocardiography showed regional wall motion abnormality in the inferior left ventricular wall. The patient was diagnosed as acute ST-segment elevation myocardial infarction due to allergic coronary vasospasm, the allergen was recognized as injection cervus and cucumis polypeptide. He had neither injected cervus and cucumis polypeptide nor suffer any attack of chest pain during the 1-year follow-up.

### Case 3

A 68 year-old male without known cardiac risk factors had suffered bronchial asthma for 2 years, aggregating for 3 months with the suspected allergen of detergent and pesticide sprays since he moved from south China to Beijing, accompanied by recurrent precordial squeezing pain, dyspnea and excessive sweating several hours after the episodes of wheezing and spontaneously resolved in 10~15 minutes. After admitted to the cardiology unit, he complained allergy to the smell of pesticide sprays and suffered chest pain for several times at 1:00~3:00 in the morning which was relieved by intravenous administration of nitroglycerin. ECG showed ST segments depression of 0.2-0.3 mv in leads V1-V6 on attack and resolution of ST segments when symptom was resolved, Holter showed non-sustained ventricular tachycardia at the time he felt dyspnea, while repeated tests of troponin I were negative. Coronary angiography was performed on the third day after admission, revealing a 90% stenosis in the proximal segment of LAD, a 75% stenosis in the middle segment of LAD and a 50% stenosis in the proximal segment of LCX respectively, and all stenosis disappeared after 200ug intracoronary administration of nitroglycerin. The patient was diagnosed as coronary vasospasm and was given oral isosorbidemononitrate and diltiazem for long-term therapy. The attacks of chest pain developed less frequently but still relapsed after wheezing, he could predict the mid-night chest pain by the preceding contact of allergen and wheezing, and experimentally taking loratadine (which is a non-sedating anti-histamine drug that inhibit the release of histamine from mast cells) could effectively prevent or alleviate the episodes of chest pain.

### Case 4

**Fig. 9:**
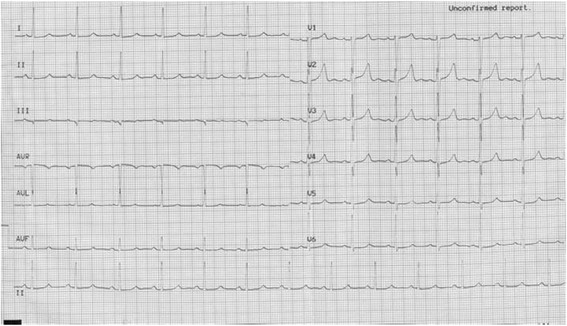
ECG on admission was normal

**Fig. 10:**
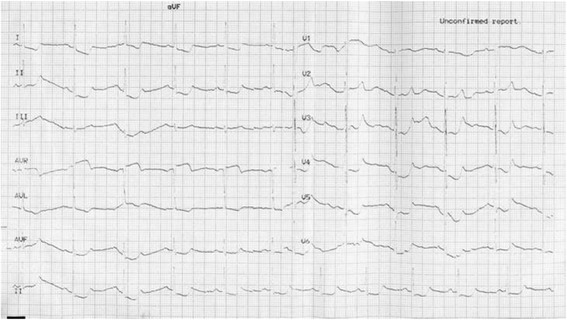
Downsloping ST segments depression of 1-3 mm in leads I, aVL, II, III, aVF, V2-V6

A 42 year-old female without history of atopy and cardiovascular risks received surgical abortion under intravenous anesthesia of Propofol in February, 2013. At 7:00 on the 7^th^ morning of the surgery, she suffered a sudden severe pressure-like pain of 10 minutes in the left subclavian and retrosternal area when she was lying in bed. After that, such pain recurrently occurred in the midnight and early morning. She was diagnosed as angina pectoris in the local hospital and given aspirin, clopidogrel, atorvastatin and nitrates, but chest pain still relapsed. After a serious attack of 15 minutes with heavy sweating at 2:00, she was sent to our hospital. On admission, her blood pressure was 150/100 mmHg, heart rate was 76 beat per minute, physical examination revealed no abnormalities. ECG on admission was normal (Fig. 9), troponin I was negative and the rate of eosinophil was 2.5%, coronary angiography revealed a plaque in the proximal segment of LAD without stenosis and other coronary arteries were normal. She was suspected as coronary vasospasm and was given mononitrate, nifedipine, aspirin, clopidogrel and atrovastatin. However, ischemic chest pain continued attacking in the morning during hospitalization. On the 3^rd^ morning after angiography, she suffer a 5-minute episode, ECG showed downsloping ST segments depression of 1~3mm in leads I, aVL, II, III, aVF and V2-V6 (Fig. 10), the symptom was relieved by one dose (0.5 mg) of sublingual nitroglycerin and ST segments recovered, whereas another two attacks occurred 2 hours and 4 hours later, respectively, with the same ECG presentation. After continuous 48-hour intravenous administration of nitroglycerin, as well as added dose of mononitrate and nifedipine before night sleep, chest pain seemed to calm down, she kept symptomless for 5 days and discharged. She occasionally suffered early morning chest pain for 5-10 minutes in the first 4 months though adhering to drugs. Interestingly, the episodes suddenly stopped when her menopause recovered in August, 2013, and her chest pain never relapsed during the 4 years follow-up.

### Case 5

**Fig. 11:**
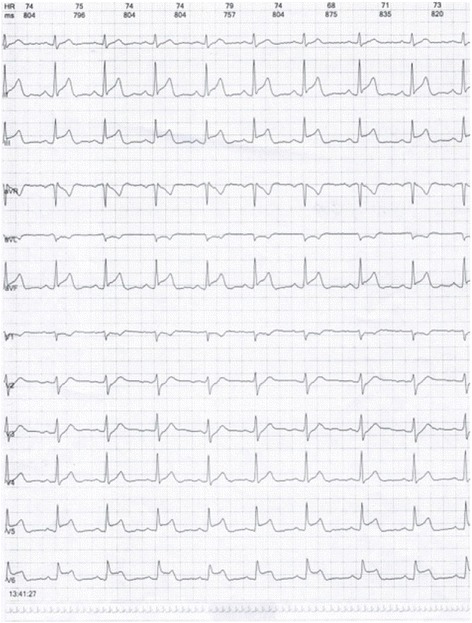
ST segment elevation for 2-3 mm in leads II, III, aVF, V5, V6 at13:40-13:42 in Holter

**Fig. 12:**
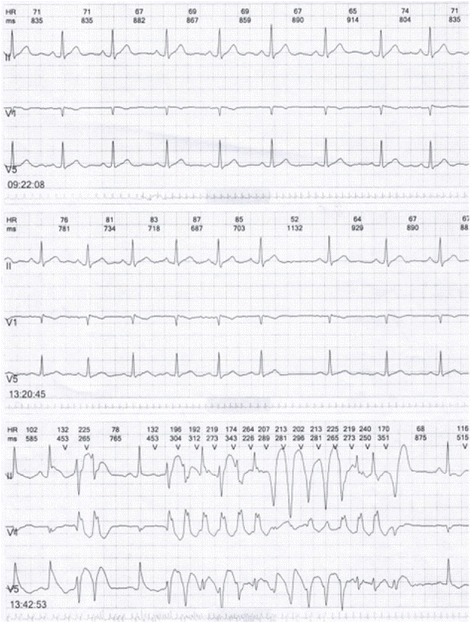
Nonsustained ventricular tachycardia at 13:42 in Holter

**Fig. 13:**
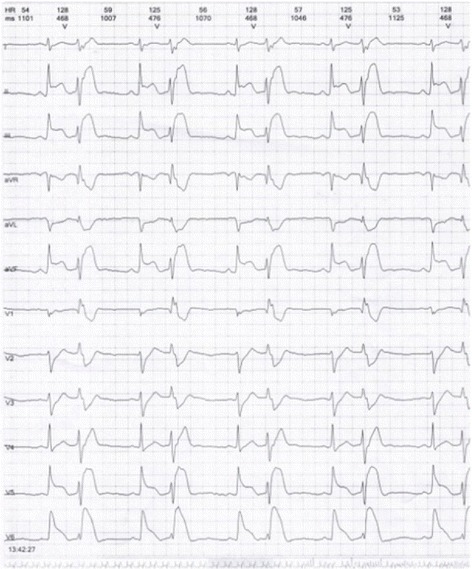
Ventricular bigeminy at 13:40 in Holter

A 45 year-old male developed flushing and urticaria 15 minutes after eating pineapple, then he felt precordial squeezing pain radiating to the left shoulder, lasting for several minutes, accompanied by palpitation and excessive sweating. He is a smoker with no other cardiac risk factors and no history of CVD, he was allergic to pineapple before but only manifested as slight itching and skin rashes which spontaneously disappeared in hours. Before this onset, he had been working day and night for 1 month with doubled tabacco smoking, though he did not smoke after eating pineapple. The patient was sent to the emergency room, on arriving, his symptoms had been relieved by sublingual nitroglycerin, his blood pressure was 120/70mmHg, pulse rate was 80 beat per minute, and ECG revealed no abnormalities while the symptom had relieved. 24h Holter (Figs. 11, 12 and 13) revealed ST segment elevation for 2~3 mm in leads II, III, aVF, V5, V6 at 13:40-13:42 (when he felt malaise, chest pain and palpitation at rest) and 20:20-20:22 (when he was driving, he felt chest pain and left arm numb), as well as ventricular bigeminy and nonsustained ventricular tachycardia at 13:20 and 13:42. He was admitted to cardiology department, blood test showed normal troponin I level and elevated eosinophil of 7.6% on the first day of admission. Coronary angiography showed subocclusion in the proximal segment of LCX with TIMI grade 3 flow, which was relieved by 3 times of 200μg intracoronary nitroglycerin. He was diagnosed as coronary vasospasm on the basis of anaphylaxis to pineapple, and received anti-vasospasm therapy of mononitrate and nifedipine as well as antiallergic agent, the eosinophil rate declined to 6.1% on the 7^th^day, chest pain didn’t relapse during hospitalization. After 12 months of mononitrate and nifedipine medication, anti-vasospasm therapy was discontinued. During 3 years follow-up, he kept free of cardiac events.

## Discussion

We report 5 rare cases of KS induced by unreported exposures or circumstances. To our knowledge, this is the first time reporting Chinese traditional herbs as the sensitizer of allergic coronary vasospasm.

The two patients had no cardiovascular risk factors and pre-existing atherosclerotic diseases, they experienced recurrent coronary vasospasm and acute myocardial infarction, accompanied by definite manifestations of anaphylaxis obviously elevated level of blood eosinophil, supporting the diagnosis of allergic coronary vasospasm based myocardial infarction. Angiography revealed no stenosis after the vasospasm relieved, indicating the diagnosis of type I KS [5].

In case 1, both asthma and the decoction were the possible culprit responsible for coronary vasospasm. Wheezing may be associated with an increased risk of coronary vasospasm, both the status of asthma and the allergic factors may cause the onset of vasospasm [6]. However, the onset of asthma was 6 months earlier than coronary vasospasm, during the recurrent episodes of wheezing in this 6 months, there were no attacks of ischemic chest pain; during the 4 years follow-up, wheezing still occurred although the chest pain had disappeared, indicating that the time window of coronary vasospasm was not consistent with the status of wheezing. On the contrary, the patient suffered skin allergy (which provided the evidence of anaphylaxis induced by herbs) as well as recurrent ischemic chest pain on the first day taking the decoction, chest pain was relieved by the discontinuation of the decoction and relapsed when the patient resume the decoction again, strongly indicating the close relationship between TCM-induced anaphylaxis and coronary vasospasm, supporting the point that the anaphylaxis induced by Chinese decoction rather than the status of asthma should responsible for the frequent episodes of coronary vasospasm. Furthermore, the status of asthma might put the patient into an exposure of hypersensitivity, and the decoction induced anaphylaxis on the basis of this hypersensitivity.

“Ma-Xing Shi Gan Tang” is a classical decoction for asthma which is boiled in water with 16 plants and animal composition. We considered the whole decoction as the inducer of KS, but it’s difficult to precisely tell which one of the 16 components or their compound (formed during boiling) should be the culprit. Ephedra (Ma-Huang) and earthworm (Di-Long) are the two main effective components in the decoction, so we analyzed these two components in detail.

Ma-Huang is a classical bronchodilator in TCM with a history of medical use for over 5000 years, ephedra is the effective constituent. In the western countries, ephedra as a mainstream treatment for asthma reached its zenith in the late 1950s, and then moved into the twilight zone with the emergence of other drug groups. Ephedra contains 1~2% of ephedrine, which is its primary active ingredient. Ephedrine is a predominantly indirectα-and β-adrenoceptor agonist for heart and blood vessels, could cause peripheral vasoconstriction and coronary vasospasm [7, 8]. In the US, there are more than 16,000 reports about the adverse events associated with ephedra-containing dietary supplements, especially acute myocardial infarction and stroke [9]. However, the procedure we manage ephedra in Chinese decoction is different from the procedure of chemical isolation and purification in western countries, the plant of ephedra (Ma-Huang) is mixed with other plants and boiled in water, so the concentration of effective constituent should be much lower, this might explain the rare reports about Ma-Huang related coronary vasospasm in Chinese medicine. In case 1, we suppose the coronary artery was hypersensitive to ephedrine and experienced anaphylaxis that lead to skin allergy and coronary vasospasm.

Di-Long, also known as common earthworm (*lumbricusterrestris),* is one of the most common component of Chinese decoction for asthma therapy. The earthworm peptides were demonstrated to regulate immune-responsiveness and relieve bronchial spasm [10], but it’s also a source of foreign protein reasonable to be a suspicious allergen. Several case reports had described patients suffering allergic angioedema, conjunctivitis, rhinitis and urticarial caused by common earthworm *L.terrestris* (used as fish baits), type I hypersensitivity mechanism was demonstrated to be responsible [11–13]. Carreñoet et al [14] recognized 1 allergen of around 15.5kDa in 13cases allergic to *L terrestris*, which provided a molecular proof for earthworm allergy. We consider earthworm as a probable culprit allergen for the coronary vasospasm in this case.

In case 2, Allergy is evidenced by anaphylactic shock and raised blood eosinophil count; the patient had no cardiac risk factors, no history of coronary heart disease and no such episodes during follow-up, while the isolated coronary event happened exactly 30 minutes after the allergic shock, which logically highly pointed to the link of allergy and coronary event. There are two reasonable explanation for this coronary event: (1) allergic vasospasm (type 1 KS) or vasospasm triggered plaque rupture (type 2 KS) might be a logically reasonable explanation. (2) However, the coronary angiography was performed 5 hours after the event, we have no direct evidence of vasospasm. Thrombus formed over a ruptured yet subcritical plaque might be another reasonable explanation, which could have dissolved after 5 hours when coronary angiography was performed, and the eosinophil raise could be linked to a reaction to the traditional medication. The anaphylactic shock and coronary vasospasm based STEMI had a definitely close relation with injection CCP. CCP is the combined extracts from deer horn and sweet melon seeds, which has gained popularity in orthopedic clinics in China, with the aim to promote fracture healing and treat osteoarthritis and rheumatoid arthritis. Till now, we have not found any reports about CCP induced anaphylaxis.

Decoction is a kind of liquid mixture made of various Chinese materia medica (CMM) including medical plants, insects and animal components under the procedure of soak, boil and steam. There are thousands of CMM in China, a decoction usually contains twenty kinds of CMM, the prescriptions are fairly different according to different patients, diagnosis or symptoms. CMM are proved to effectively modulate constriction-dilation of blood vessels, modulate oxygen reply-demand and influence the stability of plaques [15]. The functional components are proved to be the various bioactivators (proteins, alkaloids) rooted from herbs [16], which also might act as allergens and toxicants, although reports about CMM induced allergy are very rare. We suppose that allergic coronary artery spasm is not a rare condition but a rarely suspected and under-diagnosed condition, our reports may remind physicians of the linkage between ischemia chest pain and the anaphylaxis induced by TCM.

Kounis syndrome, also known as allergic angina syndrome, was described in 1991 by Kounis and Zavras [17] as “the concurrence of chest pain and allergic reactions, accompanied by clinical and laboratory findings of classical angina pectoris caused by inflammatory mediators released during the allergic insult”. Allergic angina could progress to acute myocardial infarction which was named allergic myocardial infarction [18]. The main pathophysiological mechanism is vasospasm of epicardial coronary arteries due to increased inflammatory mediators that are released during a hypersensitivity reaction.

KS should be kept in mind especially when diagnosing patients without CV risk factors and pre-existing CVD who experience acute coronary syndrome and report ingestion of a drug accompanied by symptoms of anaphylaxis. Ruptured plaques, a traditional known reason for acute coronary syndrome, may not be the explanation for this case. It seems that atopic individuals are at higher risk of acute coronary syndromes than normal people [19]. A population based study revealed a causal role of IgE in the development of cardiovascular disease [20], mast cell degranulation inhibitors might prevent acute thrombotic events [21], this may explain why administration of clopidogrel helped to cease the episodes of ischemic chest pain in our cases.

Several pathophysiological mechanisms have been described to explain the involvement of the heart in anaphylactic reactions. The existence of mastocytes in heart tissue and their participation in the anaphylactic reaction that triggers coronary vasoconstriction, dysfunctional ventricular contractility and blockade of atrioventricular conduction is well known. These abnormalities are attributed to the release of inflammatory mediators such as histamine, thrombin, prostaglandins, leukotrienes and platelet activation factors, as well as the release of rennin during episodes of anaphylaxis and its involvement [22]. The factors released from mastocytes and other interacting inflammatory cells during the anaphylactic activation and manifests as vasospastic angina, acute myocardial infarction and stent thrombosis. A subset of platelets bearing, in their surface, FCγRI, FCγTII, FCεRII receptors are also involved in this activation cascade [23].

In Case 3, KS is a reasonable explanation, but not the only. We consider two explanations: (1). The patient showed bronchial hypersensitivity to the exposure of pesticide sprays, followed by episodes of coronary vasospasm, it’s reasonable to consider that there might be systemic hypersensitivity involved in both bronchus and coronary artery. Bronchial and myocardial involvement with early severe bronchoconstriction and vasospasm-induced coronary blood flow reduction manifesting as KS respectively should be always considered. Combined bronchoconstriction with interstitial edema and tissue suppression from arterial involvement and peripheral vasodilatation, perhaps, occur simultaneously [24]. (2). The coronary artery is not a direct target organ of hypersensitivity, the coronary spasm might be secondary to smooth muscle contraction reflex caused by the irritation of the bronchial epithelium by molecules, the bronchial smooth muscle contraction reflex induced the epithelium derived inflammatory molecules accumulation, and cause coronary vasospasm through this pathway.

In Case 4, after surgical abortion under anesthesia of propofol, a childbearing woman experienced refractory angina, which was highly suspected as coronary vasospasm evidenced by classical clinical manifestations of angina and nearly normal coronary artery in angiography. Several case reports described transient coronary vasospasm based cardiac events under the administration of Propofol [25], but in our case, the first onset of angina occurred 7 days after the administration of propofol, and the following refractory relapse had no relation with propofol, indicating the little causality between propofol administration and angina. Another explanation might be as below: the surgery had injured her endometrium and disturbed the ovarian function, evidenced by the significant delay of menstruation recovery. Because endogenous estrogens are involved in the regulation of vascular tone and protect premenopausal women from development of coronary disease [26, 27], the post-abortion disorder of estrogen/progestin might lead to the imbalance between coronary vasoconstriction and vasodilation, therefore cause refractory vasospastic angina. The refractory vasospasm occurred synchronously with the loss of menstruation and spontaneously disappeared when the ovarian function returned, supporting our assumption that the exposure to disturbance of ovarian function induced by surgical abortion might be the inducer of KS.

Case 5 displayed a patient suffering ST-elevated ACS and malignant ischemic ventricular arrhythmia due to allergic coronary vasospasm, the allergen seemed to be pineapple, to which he only had slight skin allergy history, it’s interesting why severe coronary vasospasm was involved this time. One reasonable explanation may be that the anaphylaxis exploded on the basis of “preconditioning” of the exposure to tabacco smoking and stress, causing explosive release of inflammatory mediators and trigger anaphylactic reaction in the heart. In fact, stress could precipitate allergies and trigger coronary mast cells leading to cardiac events [28]. Though smoking exposure is a recognized risk factor for coronary vasospasm in susceptible individual [29], this patient did not suffer vasospastic angina when he doubled smoking, whereas he did not smoke when the pineapple-induced angina broke out, implying that smoking was not the direct inducer of vasospasm. Thus neither pineapple-induced anaphylaxis nor smoking independently triggered vasospasm events. Our explanation is: the exposure of smoking and stress provided “preconditioning” status for the realization of anaphylactic coronary vasospasm induced by pineapple.

## Conclusion

Allergic coronary vasospasm is not rare but rarely recognized and under-diagnosed. It is necessary to recognize KS and various inducers, especially for the patients suffering refractory vasospatic cardiac attacks concentrating in special periods. Blood test of eosinophil might contribute to diagnose KS and anti-allergic agents might be helpful for controlling KS attacks.

## Abbreviations

ECG: Electrocardiogram
KS: Kounis syndrome
STEMI: ST-segment elevation myocardial infarction
TCM: Traditional Chinese medicine
